# Insights into the prognostic value of DJ-1 and MIB-1 in astrocytic tumors

**DOI:** 10.1186/1746-1596-8-126

**Published:** 2013-07-31

**Authors:** Rasha M Abd El Atti, Hoda H Abou Gabal, Wesam M Osman, Amr S Saad

**Affiliations:** 1 Department of Pathology, Faculty of Medicine, Ain Shams University, Cairo, Egypt; 2 Clinical Oncology, Faculty of Medicine, Ain Shams University, Cairo, Egypt

**Keywords:** DJ-1, MIB-1, Immunohistochemistry, Prognosis, Astrocytoma

## Abstract

**Background:**

The histological grade is the gold standard for the evaluation of prognosis of astrocytic tumors. Nevertheless, morphologic criteria are not always accurate prognostic indicators.

**Aim:**

The research investigates the expression of MIB-1 and DJ-1 in different grades of astrocytomas and evaluates the possible prognostic role of DJ-1 in these tumors in relation to other prognostic parameters including the MIB-1 labeling index.

**Materials and methods:**

Immunohistochemical expression of MIB-1 and DJ-1 was evaluated in 111 samples of astrocytic tumors comprising 28 diffuse astrocytomas, 38 anaplastic astrocytomas and 45 glioblastomas. The univariate survival analysis was done using the Kaplan-Meier method and the multivariate survival analysis was done using Cox proportional hazard model.

**Results:**

The statistical analysis revealed a significant correlation between each of DJ-1 and MIB-1 and the histological grade of astrocytomas. The univariate analysis showed that high grade, high DJ-1 score and MIB-1 labeling index ≥ 10.1 were associated with poor survival. Multivariate analysis for all the studied astrocytomas proved the independent prognostic significance of the histological grade and DJ-1 score. Meanwhile, the multivariate analysis for each grade emphasized that DJ-1 was the only independent prognostic indicator in high-grade astrocytomas.

**Conclusion:**

This study emphasized the effectiveness of high DJ-1 expression in predicting poor survival of astrocytoma patients, when compared to MIB-1. DJ-1 could be particularly important in cases with discrepancies between the morphologic criteria and clinical parameters.

**Virtual slides:**

The virtual slide(s) for this article can be found here: http://www.diagnosticpathology.diagnomx.eu/vs/1070116023943146

## Introduction

Tumor grading is one of the most significant predictors of the clinical outcome. Astrocytomas, the most common primary intracerebral tumors are not an exception in this respect. The high-grade astrocytomas are usually associated with poor prognosis. However, the histological differentiation may be confusing in some cases, especially when small stereotactic guided needle biopsies are available. Therefore studies have employed additional diagnostic and prognostic measures for predicting clinical outcome and survival [1].

Many studies have focused on the proliferative activity in astrocytomas especially ki-67/MIB-1 labeling index (LI). MIB-1 antibody is an IgG1 class monoclonal antibody which recognizes a core antigen present in the nuclei of the cells in the G1, S, G2 and M phases of the cell cycle, but is not expressed in the resting phase, G0 [2]. Importantly, the MIB-1 LI is not a component of the WHO grading scheme for glial neoplasms [3]. However many investigations have demonstrated a significant positive correlation between MIB-1 indices & histological grade and have shown that higher MIB-1 LI is associated with shorter survival [4,5].

DJ-1 (PARK-7) is a protein with antioxidative stress and antiapoptotic properties. The antioxidative role of DJ-1 results from its participation in mitochondrial stabilization upon exposure to an oxidative stress [6,7].

The antiapoptotic ability of DJ-1 is attributed to several mechanisms: inhibition of phosphatase and tensin homolog (PTEN) mediated inhibition of the phoshoinositide 3-kinase (PI3K)/AKt antiapoptotic pathway. DJ-1 interacts with and inhibits the function of death-associated protein 6 (Daxx) which binds to apoptosis signal-regulating kinase1 to promote apoptosis [8]. DJ-1 also induces Nrf2 stability, which in turn activates the transcription of antioxidant and detoxification enzymes [6]. More recently DJ-1 has been shown to have a direct role in inhibiting apoptosis by decreasing the expression of Bax and inhibits caspase activation [9].

This strong antiapoptotic function of DJ-1 may have a significant impact on neoplastic transformation and tumor proliferation in humans, a possibility that is supported by DJ-1 expression in many cancers. DJ-1 has been reported to be over expressed in lung, breast, pancreas, esophageal and urinary bladder cancers [10-12].

As regards the astrocytic tumors, DJ-1 expression may be particularly important since it is prominently expressed in reactive astrocytes in both acute and chronic forms of human neurodegenerative diseases [13,14].

The important role of DJ-1 as an oncogene suggests that it may be a possible prognostic indicator in patients with astrocytomas.

This study investigates the possible role of DJ-1 in the progression and prognosis of astrocytic tumors and emphasizes the relationship between DJ-1 and other important prognostic factors including the MIB-1 LI.

## Materials and methods

A retrospective study included a total number of 111 formalin-fixed and paraffin embedded primary supratentorial astrocytomas that were surgically resected (attempt of debulking) at the neurosurgery department of Ain Shams University Hospitals, Cairo, Egypt during the period from January 2005 until January 2008. The clinical data were obtained from the patients’ medical records and included age, sex and postoperative survival data.

The patients in this study included 74 males (66.7%) and 37 females (33.3%) with a mean age of 48.5 ± 10.8 (range: 12–71). The follow up period was the interval from the date of initial diagnosis until the date of death, or the completion of the research. During the follow up period, 92 patients (82.9%) died of the disease, while 19 patients (17.1%) were still alive by the end of the study with median survival 20m (range: 3m-68m). The study was carried out with full local ethical approval. This study gained the institutional review board approval from Research Ethical Committee at Faculty of Medicine, Ain Shams University.

Pathology slides from all resected tumor specimens were reviewed by each author to confirm the diagnosis of each grade of the studied astrocytoma cases using the established criteria of the WHO classification of the brain tumors [3]. The availability of sufficient suitable material for the immunohistochemical studies was essential.

The astrocytoma cases were classified into 28 diffuse astrocytomas (grade II), 38 anaplastic astrocytomas (gradeIII) and 45 glioblastomas (GBMs) (grade IV). The grading of necrosis in GBMs was based on the amount observed in magnetic resonance imaging (MRI) scans. The following grading system was applied: grade 0, no necrosis; grade 1, amount of necrosis is < 25% of the tumor volume; grade 2, amount of necrosis is between 25% and 50% of the tumor volume; grade 3, amount of necrosis is > 50% of the tumor volume [15]. Grade 1 necrosis, grade 2 necrosis and grade 3 necrosis were observed in 42.2% (19/45), 40% (18/45) and 17.8% (8/45) of the studied GBMs respectively.

### Immunohistochemistry

All 111 astrocytoma samples were subjected to immunohistochemical staining with MIB-1 and DJ-1 antibodies. The paraffin embedded tissue sections were deparaffinized in xylene and rehydrated through absolute alcohol. Antigen retrieval in citrate buffer (pH9 Lab vision cat#AP9003) was used after the sections were treated in a microwave at 8w for 5–6 min, then at 3w for 10 min; the sections were then left to cool for 20 min. Peroxidase and protein block were done. Following this, slides were incubated overnight with each of the primary antibodies at room temperature using MIB-1 antibody (rabbit monoclonal antibody, 7 ml, Lot. No. 1210212B, Cell marque, CA, USA) and DJ-1antibody (mouse monoclonal antibody, dil (1:20), Cat # 37–8800, Cell marquee, CA, USA), followed by rinsing in PBS (pH7.6) This was followed by the secondary biotin conjugated antibody for 1 hour and finally the peroxidase conjugated streptavidin for another hour. Diaminbenzidine tetrachloride (DAB) (freshly prepared) was added for 25 min, then counterstained in Harris Hematoxylin, followed by dehydration, clearing and mounting. Positive control for MIB-1 antibody was breast carcinoma, while positive control for DJ-1 was prostatic and kidney tissue. Negative controls were done by excluding the primary antibody and its replacement with a non-immune antibody.

### Interpretation of immunohistochemical staining of MIB-1

The results of immunohistochemical staining were assessed by each author and a consensus regarding controversial cases was reached at a multiheaded microscope.

MIB-1 imunostaining was evaluated in the fields of maximal labeling. The MIB-1 LI was the number of MIB-1 labeled tumor nuclei expressed as a percentage of the total number of tumor nuclei counted. A total of at least 1000 nuclei was counted in each case [1].

### Interpretation of immunohistochemical staining of DJ-1

The score of DJ-1 cytoplasmic immunoreactivity was assigned based on the intensity of staining. The score was ranging from 0 to 3 (i.e. 0 = negative, 1 = weak intensity, 2 = moderate intensity, 3 = strong intensity) [10].

### Statistical analysis

Continuous variables are expressed as mean or median and Standard Deviation. Categorical variables are expressed as frequencies and percents. The ROC Curve (receiver operating characteristic) was used to evaluate the Sensitivity and specificity of MIB in prediction of mortality among cases. *ANOVA* Test was used to assess the statistical significance of the difference between more than two study group mean. Chi square and Fisher’s exact test were used to examine the relationship between Categorical variables. Spearman’s correlation was used to assess the correlation between grade and DJ. Survival rates were estimated and graphed using the Kaplan-Meier method. Log rank test was used to compare time-to-event variables by levels of a factor variable. Cox Regression was used for modeling the time to a specified event, taking into consideration the values of other given variables. A significance level of *P <* 0*.*05 was used in all tests. All statistical procedures were carried out using SPSS version 15 for Windows (SPSS Inc, Chicago, IL, USA).

## Results

### Immumohistochemical results

#### ***MIB-1 expression and its associations in the studied astrocytoma cases***

**Figure 1:**
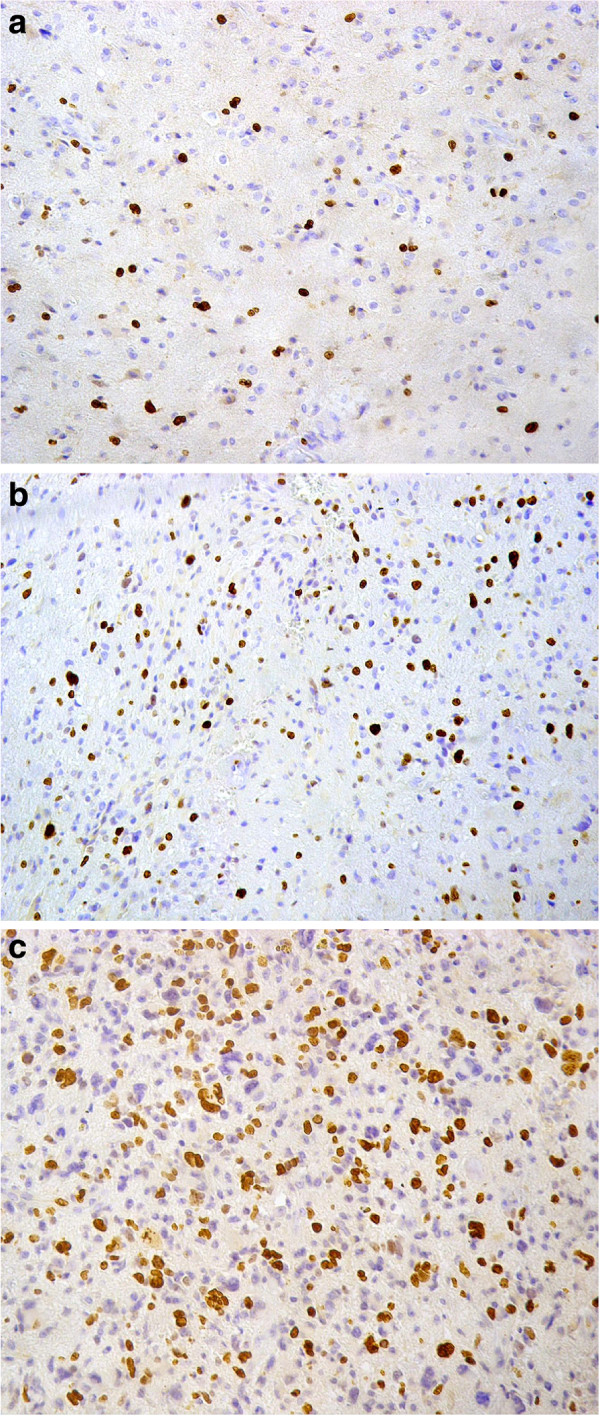
**MIB-1 labeled nuclei in astrocytomas. a**: In diffuse astrocytoma (MIB-1x200). **b**: In anaplastic astrocytoma (MIB-1x200). **c**: In glioblastoma (MIB-1x200).

The mean MIB-1 LI of diffuse astrocytomas, anaplastic astrocytomas and GBMs were (4.26 ± 2.43), (13.54 ± 2.82) and (26.43 ± 5.18) respectively. There was a significant difference between the diffuse astrocytomas, the anaplastic astrocytomas and the GBMs as regard the mean MIB-1 LI (F = 295.9, P = 0.0001). The Post hoc test (LSD) revealed a significant difference in the mean MIB-1 LI between the diffuse and anaplastic astrocytomas (P = 0.001), and the anaplastic astrocytomas and GBM cases (P = 0.001) (Figure 1a, 1b and 1c).

**Figure 2:**
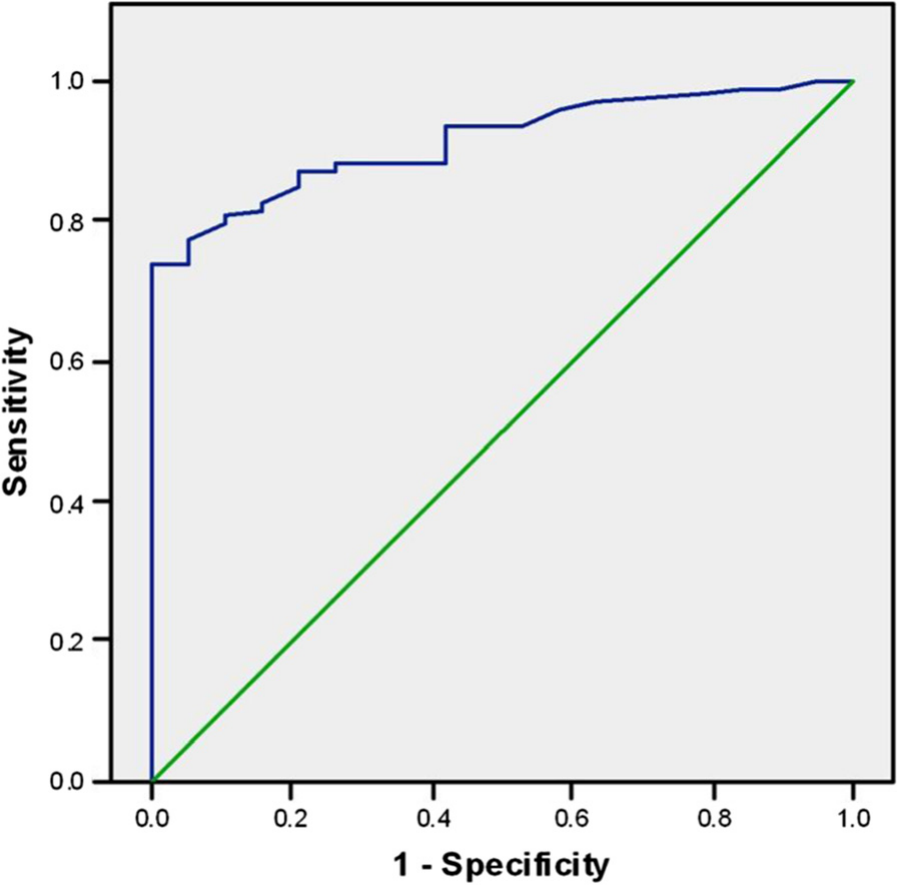
ROC curve to evaluate the sensitivity and specificity of MIB in prediction of mortality.

The MIB-1 LI of 10.1 was considered to be a highly significant prognostic cut off value, as MIB-1 LI ≥ 10.1 could predict mortality with 81.5% sensitivity, 84.2% specificity, 96.1% positive predictive value (PPV), 48.5% negative predictive value (NPV), 95% CI = (0.86-0.966), AUC = 0.913, LR + = 5.163 and LR- = 0.219 (Figure 2).

**Figure 3:**
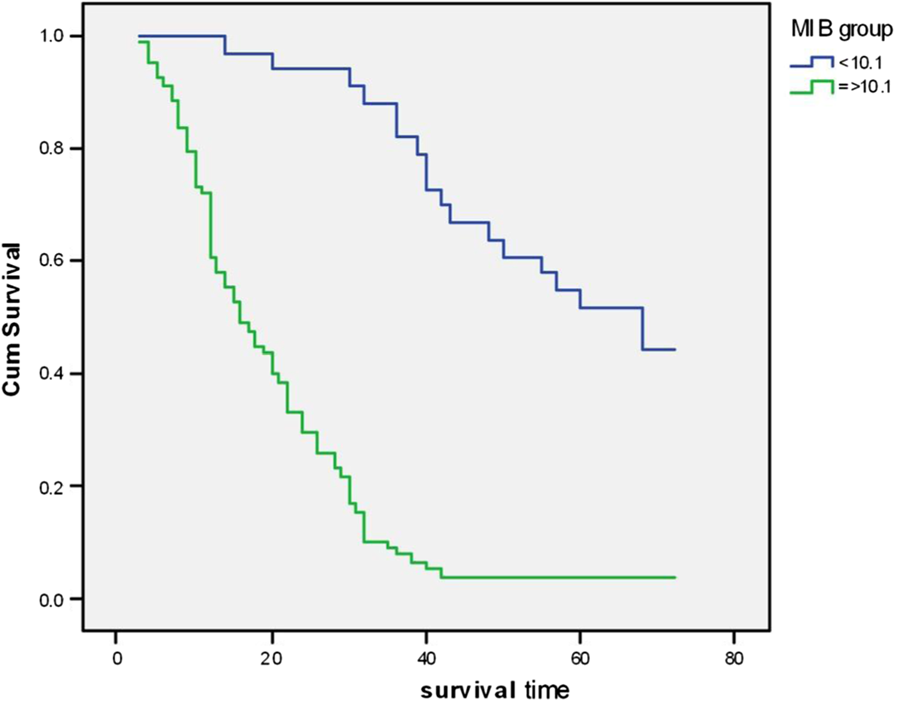
Kaplan-Meier overall survival curves for tumors with MIB-1 LI < 10.1 and MIB-1 LI ≥ 10.1.

Kaplan- Meier survival showed that the astrocytoma cases with MIB-1 LI ≥ 10.1 were associated with shorter median survival (16m ± 2.207, 95% CI: 11.674 -20.326), when compared to those cases with MIB-1 LI < 10.1 which were associated with longer median survival(68m ± 9.785, 95% CI: 48.822 -87.178). Therefore, there was a high statistically significant difference between the two groups as regards the median survival (log rank = 54.87, P = 0.0001) (Data not tabulated) (Figure 3).

### DJ-1 expression and its associations in the studied astrocytoma cases

The DJ-1 staining pattern in the tumor cells was almost cytoplasmic with minimal or no nuclear staining.

DJ-1 positive cytoplasmic expression was compiled in 92.8% of all cases (103/111) [score 1 (15.3%) (17/111), score 2 (28%) (31/111), and score 3 (49.5%) (55/111)], while the remaining 8 cases (7.2%) showed negative DJ-1 expression (score 0).

**Figure 4:**
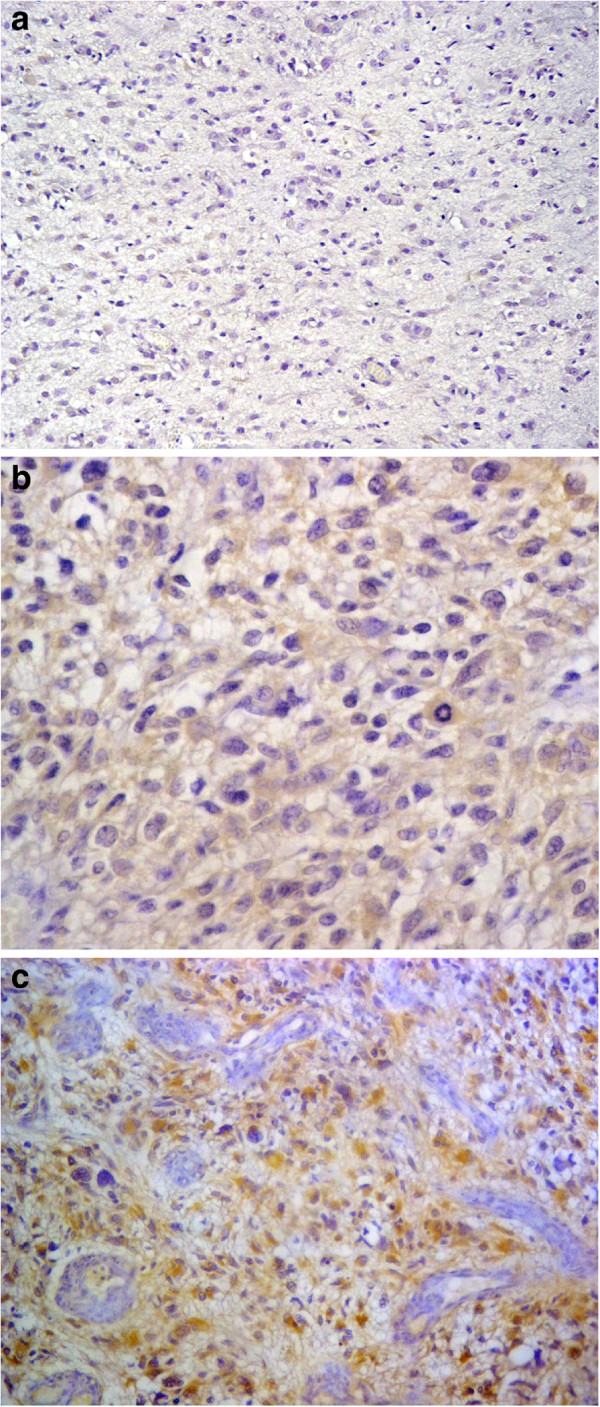
**DJ-1 expression in astrocytomas. a**: Weak DJ-1 staining intensity in diffuse astrocytoma (DJ-1x200). **b**: Moderate DJ-1 staining intensity in anaplastic astrocytoma ( DJ-1x 400). **c**: Strong DJ-1 staining intensity in glioblastoma (DJ-1x200).

Cytoplasmic immunostaining of the tumor cells showed a tendency to decrease in intensity with a reduction in the aggressiveness of the tumors, this was statistically evident by the high significant direct correlation between DJ-1 staining intensity and the histological grade (Rho = 0.815, P = 0.0001). The results disclosed that 88.9% of the GBMs showed DJ-1 intensity (score 3), whereas 60.7% of the diffuse astrocytoma cases exhibited DJ-1 staining intensity (score 1) (Table 1). On further comparison, the post hoc test (LSD) showed that there was a high significant difference between diffuse astrocytomas and anaplastic astrocytomas, and anaplastic astrocytomas and GBMs as regard DJ-1 staining intensity (P = 0.0001) (Figure 4a, 4b and 4c).

**Table 1:** Relation between DJ-1 and clinicopathological variables in the studied astrocytomas

		**DJ-1 0**		**DJ-1 1**		**DJ-1 2**		**DJ-1 3**		***X*2**	**P**	**Sig**
		**N**	**Row %**	**N**	**Row %**	**N**	**Row %**	**N**	**Row %**			
**Grade**	Grade II	8	28.6%	17	60.7%	3	10.7%	0	.0%	113.813**	.0001	HS
	Grade III	0	.0%	0	.0%	23	60.5%	15	39.5%			
	Grade IV	0	.0%	0	.0%	5	11.1%	40	88.9%			
**Sex**	Male	5	6.8%	13	17.6%	25	33.8%	31	41.9%	6.151*	.104	NS
	Female	3	8.1%	4	10.8%	6	16.2%	24	64.9%			
**Age group**	<50 years	6	10.9%	11	20.0%	18	32.7%	20	36.4%	8.19**	0.04	HS
	≥50 years	2	3.6%	6	10.7%	13	23.2%	35	62.5%			
**MIB group**	<10.1	8	24.2%	17	51.5%	6	18.2%	2	6.1%	78.16*	.0001	HS
	≥10.1	0	.0%	0	.0%	25	32.1%	53	67.9%			
**Necrosis**	1	0	0%	0	0%	2	10.5%	17	89.5%	1.186**	0.707	NS
	2	0	0%	0	0%	3	16.7%	15	83.3%			
	3	0	0%	0	0%	0	0%	8	100%			

**Fisher’s Exact Test.

*Chi-Square Tests.

Also a significant direct correlation was found between DJ-1immmunoreactivity and MIB-1 LI (Rho = 0.832, P = 0.0001) and between DJ-1 and age (Rho = 0.404, P = 0.0001).

The relation between DJ-1 expression and the clinicopathological variables in the astrocytoma patients was emphasized in Table 1.

**Figure 5:**
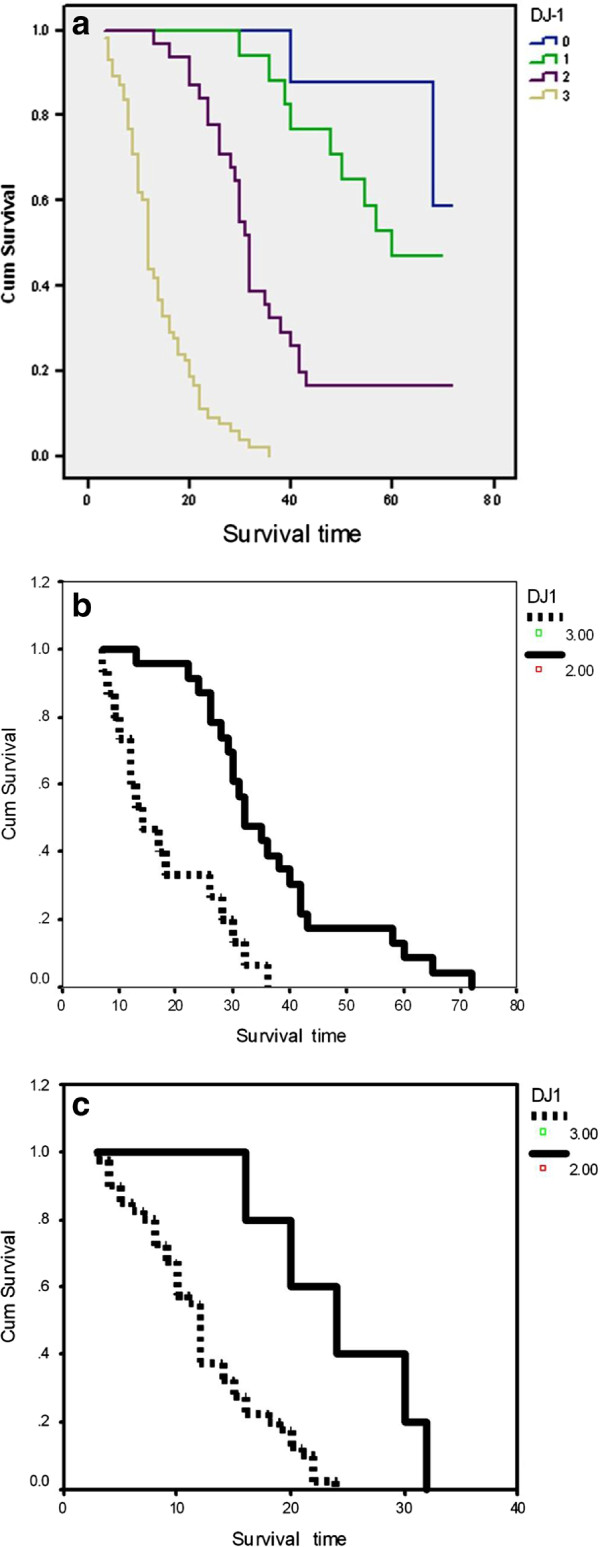
**Kaplan-Meier overall survival curves according to DJ-1 level. a**: In all astrocytoma cases. **b**: In anaplastic astrocytoma cases. **c**: In glioblastoma cases.

In all astrocytoma cases, the univariate analysis of overall survival showed high DJ-1 expression to be a poor prognostic factor (Table 2) (Figure 5a). Other poor predictors of survival on univariate analysis included old age group (> 50), female gender, and high tumor grade (Table 2).

**Table 2:** Univariate analysis of the prognostic factors in relation to overall survival in the studied patients

	**Median survival (months)**			**Log rank**	**P value**
	**Estimate**	**Std error**	**95% CI**		
**Age**					
<50	32.000	3.178	25.771-38.229	11.874	0.001(HS)
≥50	16.000	2.137	11.812-20.188		
**Gender**					
Male	26.000	3.441	19.256-32.744	4.591	0.032(S)
Female	17.000	4.054	9.055-24.945		
**Grade**					
II	68.000	-------	-----	94.619	0.0001(HS)
III	29.000	2.055	24.973-33.027		
IV	12.000	0.476	11.067-12.933		
Necrosis**					
1	15.000	4.353	6.468-23.532	2.081	0.353(NS)
2	12.000	1.034	9.973-14.027		
3	11.000	0.943	9.152-12.848		
**DJ-1**					
0	66.833*	3.710	59.561-74.105	110.349	0.0001(HS)
1	60.000	------	------		
2	32.000	1.017	30.007-33.993		
3	12.000	0.409	11.199-12.801		

*Mean.

**Necrosis is done for grade IV patients only.

On multivariate survival analysis using Cox regression, the histopathological grade and the level of DJ-1 staining intensity had independent prognostic significance, while the MIB-1 LI, age and gender lost their significance (Table 3).

**Table 3:** Multivariate cox regression model of overall survival among all cases

	**HR**	**P**	**Sig.**	**95.0% CI for HR**	
Age	1.011	.355	NS	.988	1.034
Female	1.202	.419	NS	.769	1.879
Grade*		.005	**HS**		
Grade II	.220	.551	NS	.084	3.745
Grade III	.320	.001	**HS**	.196	.679
MIB >10.1	2.661	.077	NS	.899	7.870
DJ 1**		.0001	**HS**		
DJ1(1)	2.561	.230	**NS**	.551	11.895
DJ1(2)	5.396	.093	**NS**	.754	38.621
DJ1(3)	28.978	.002	**HS**	3.618	232.094

*Grade IV reference.

**DJ0 reference.

When DJ-1 was analyzed as a possible prognostic factor in each grade of the studied astrocytomas, the Kaplan- Meier analysis proved that the anaplastic astrocytomas showing score 3 DJ-1 were associated with shorter survival (14m), when compared to the anaplastic astrocytomas with score 2 DJ-1 which were associated with longer survival (32m) (Log rank = 17.6, P = 0.0001) (Figure 5b). On the other hand the GBMs exhibiting score 3 DJ-1 were associated with shorter survival (12m) than those with score 2 DJ-1 (24m) (Log rank = 9.5, P = 0.002) (Figure 5c). Nevertheless, in diffuse astrocytomas, DJ-1 was not proved to significantly affect the overall survival (Log Rank = 3.316, P = 0.191).

Multivariate survival analysis was also performed to study the effect of the different factors including age, gender, DJ-1, MIB-1 LI and necrosis (in GBMs only) on the overall survival in each grade of astrocytomas. The results showed that DJ-1 was the only independent prognostic factor in the anaplastic astrocytomas [Cox regression, enter method, score 2 DJ-1 versus score 3 DJ-1, HR = 0.217, 95% CI (0.098- 0.48), P = 0.0001) and in the GBMs [Cox regression, backward method, score 2 DJ-1 versus score 3 DJ-1, HR = 0.192, 95% CI (0.057- 0.653), P = 0.008]. However, DJ-1 lost its independent association with survival in diffuse astrocytomas (P = 0.197).

## Discussion

The gold standard for diagnosis of astrocytic tumors is the histological examination of abundantly sampled tissue. Therefore, histopathology should be used in combination with patient’s clinical history, the neurosurgical suggestions and neuroradiologic findings to ensure the greatest accuracy in the diagnosis of such tumors [16]. Histological grading is well accepted for evaluating the prognosis of astrocytoma patients. It is important to differentiate between different grades of astrocytomas, because their clinical management differs. A number of studies have shown that high histological grade (based on increased cellularity, nuclear atypia, mitotic activity and necrosis and/or microvascular proliferation) is highly correlated with decreased survival [17,18]. Our results also revealed that high histological grade was associated with decreased overall survival and proved its independent prognostic significance.

However, the morphologic criteria are not always accurate prognostic indicators. In some instances, the histopathology diagnoses a certain grade of astrocytoma, whereas other parameters such as the clinical and neuroimaging features indicate a more advanced grade.

Therefore, recent researches investigated the expression of novel prognostic biomarkers in astrocytic tumors that could lead to an accurate classification and effective treatment. For instance, the hypermethylation status of the epidermal growth factor receptor (EGFR) and methyl-guanine-DNA methyltransferase (MGMT) could play a role in glioma progression [19]. In addition, Gulati et al. [20] indicated the adverse prognostic effect of C-erbB2 (a member of EGFR family) overexpression in anaplastic astrocytomas. Other relevant markers are the proliferation markers as minichromosome maintenance protein2 (Mcm2), survivin, mitosin and Ki67/MIB-1 [21,22].

MIB-1 LI is one of the cell proliferation markers that had been extensively employed by the histopathologists in conjunction with the traditional morphologic variables. It supports better determination of the histological grade and consequently the prognosis of astrocytoma patients [23,24].

Most of the studies have found significant differences in MIB-1 labeling indices between low and high grade astrocytomas [5,25,26]. In support to the previous studies, our results showed a significant direct correlation between MIB-1 LI and the histological grade. A significant difference was detected between the diffuse and the anaplastic astrocytomas, and the anaplastic astrocytomas and GBMs. However, Hsu et al. [25] and Rodriguez-Pereira et al. [27] could not find a significant difference between the anaplastic astrocytomas and GBMs as regards MIB-1 LI.

The majority of the previous studies proposed several prognostic MIB-1 cut off values that ranged from 1.5% to 15.3% [4,28-30]. This wide range makes the interpretation and comparisons between the different results difficult. Nevertheless, a MIB-1 LI greater than 10 may serve as a commonly used value to express a more aggressive phenotype of astrocytomas.

The results of our study succeeded in identifying a MIB-1 cut off value of 10.1 for distinguishing astrocytomas with good prognosis from cases with poor prognosis. The univariate analysis revealed a significant reduction in the overall survival of astrocytoma patients with MIB-1 LI ≥10.1. However this significant association with survival was lost in multivariate analysis after adjustment of other possible prognostic factors as age, gender, histological grade and DJ-1 protein expression.

Though some studies claimed that MIB-1 LI is an independent prognostic variable [5,31-33], others found it significant only in univariate analysis in concordance with our results [4,27,34].

The present study shows that MIB-1 LI could not also provide an independent role for predicting survival when Cox regression analysis was carried out for each grade of astrocytoma. This finding agreed with the results of McKeever et al. [35], Korshunov et al. [36] and Moskowitz et al. [37] which could not establish any prognostic role for MIB-1 LI on Cox regression analysis in diffuse, anaplastic astrocytoma and GBMs respectively. On the other hand, Giannini et al. [5] and Hsu et al. [25] found that MIB-1 LI was an independent prognostic indicator in the diffuse and anaplastic astrocytomas. Also Wakimoto et al. [33] confirmed the independent adverse effect of MIB-1 LI on survival in high-grade astrocytomas only.

There are many factors responsible for such a variation in MIB-1 labeling indices in many studies. The variability in tissue processing, immunohistochemical procedures and the different quantitation methods, make it difficult to determine a certain cut off value for clinical use. Also, the degree of inter-observer variability affects the clinical usefulness of such cut off values.

In the view of seeking additional prognostic markers in different grades of astrocytomas and owing to the well established anti-apoptotic and cell survival function of DJ-1 protein, we studied the applicability of using DJ-1 as a survival predictor marker in the studied cases of astrocytomas.

Similar to our study, Junn et al. [38] demonstrated that DJ-1 is present mostly in the cytoplasm of the tumor cells and to a lesser extent in the nucleus. They reported that on oxidative stress, DJ-1 translocates to the nucleus and mitochondria, and that mitochondrial DJ-1 appears to be primarily responsible for protection against oxidative stress.

The current results revealed a strong positive correlation between the cytoplasmic staining intensity of DJ-1 and the histological grade of astrocytomas.

Hinkle et al. [39] was the first to show that DJ-1 cytoplasmic immunoreactivity was strong in GBMs, while Miyajima et al. [40] conducted the leading study which confirmed that DJ-1 is localized in the cytoplasm in addition to the nucleus of tumor cells of astrocytomas and that the nuclear DJ-1 was inversely correlated with WHO grading.

In addition to the direct correlation between DJ-1 and grading, our results also demonstrated a strong association between DJ-1 and MIB-1 LI in the studied cases of astrocytomas. This supports the role of DJ-1 in neoplastic transformation and tumor proliferation, which is evident in several human cancers. This also reveals the molecular mechanisms by which DJ-1 is involved in cancer cell survival and aggressiveness of tumors [41].

In our set of 111 astrocytoma cases, increased cytoplasmic staining intensity of DJ-1 was associated with shortened overall survival upon univariate analysis. Furthermore, high DJ-1 expression was the only independent prognostic factor affecting survival in combination with histological grade. This was contradicted by Miyajima et al. [40] who concluded that reduced nuclear DJ-1 expression was associated with shorter survival, whereas there was no correlation between the level of DJ-1 cytoplasmic expression and the prognosis of their cohort of astrocytoma patients. This controversy may result from the variation in the subcellular localization of DJ-1 and consequently DJ-1 might have different functions in the different cellular compartments.

When the astrocytoma cases were stratified according to WHO grading system, a significant association between DJ-1 staining intensity and the patient’s overall survival was observed in high-grade astrocytomas (anaplastic astrocytomas and GBMs). In anaplastic astrocytomas, strong DJ-1 staining (score 3) was able to identify patients with shorter survival than those exhibiting moderate DJ-1 staining (score 2). Also the variation in DJ-1 staining intensity stratified the GBM patients into a group with better survival (score 2 DJ-1) and a group with worse survival (score 3 DJ-1). However, DJ-1 was not significantly associated with survival in the cohort of diffuse astrocytoma patients.

The differential expression of DJ-1 particularly in anaplastic astrocytomas may have a future significance in identifying subclasses that are more aggressive or more likely associated with worse prognosis. This could be particularly important in cases where histopathology reveals lower grades of astrocytoma, while other factors indicate more malignant phenotypes. The effect on patient’s overall survival in high grade astrocytomas was restricted to DJ-1 rendering it a poor prognostic indicator independent of other factors including MIB-1 LI.

Therefore, this study emphasizes the adverse prognostic role of high DJ-1 expression in astrocytoma patients. It was the first, to the best of our knowledge, to set up a statistical survival analysis in which DJ-1 has a more valuable role in predicting survival when compared to MIB-1 especially in high grade astrocytomas.

MIB-1 is an important clinical marker in astrocytomas. Nevertheless, several shortcomings make it difficult to standardize a proliferation index for prognostic purposes. Therefore future studies, on a larger scale, are recommended in a trial to apply DJ-1 protein as a possible adjunct or even an alternative to MIB-1 immunohistochemistry especially in the histological borderline cases such as those at the grade II-III border and grade III-IV border.

In conclusion, though the histological grade still appears to be the best guideline to prognosis in astrocytoma patients, in many instances, there is dissociation between the morphologic criteria and clinical parameters. Therefore, immunohistochemical procedures using DJ-1 might serve as a very useful supporting tool to the histopathological diagnosis.

### Consent

Written informed consent was obtained from the patient for the publication of this report and any accompanying images.

## Abbreviations

LI: Labeling index; GBM: Glioblastoma; MGMT: Methyl-guanine-DNAmethyltransferase; Mcm2: Minichromosome maintenance protein2; EGFR: Epidermal growth factor receptor.

## Competing interests

The authors declare that they have no competing interests.

## Authors’ contributions

RMA conceived, designed and coordinated the study, reviewed the histological diagnosis, evaluated immunohistochemistry, performed the statistical analysis, carried out photographing and drafted the manuscript. HHA reviewed the histological diagnosis, evaluated immunohistochemistry, participated in the study design and helped to draft the manuscript. WMO participated in the sequence alignment, performed data collection, reviewed the histological diagnosis, evaluated immunohistochemistry and critically reviewed the manuscript. ASS performed survival data collection. All authors read and approved the final manuscript.
